# Simiao Decoction Alleviates Gouty Arthritis by Modulating Proinflammatory Cytokines and the Gut Ecosystem

**DOI:** 10.3389/fphar.2020.00955

**Published:** 2020-06-24

**Authors:** Xiaoying Lin, Tiejuan Shao, Lin Huang, Xianghui Wen, Mingzhu Wang, Chengping Wen, Zhixing He

**Affiliations:** College of Basic Medical Science, Institute of Basic Research in Clinical Medicine, Zhejiang Chinese Medical University, Hangzhou, China

**Keywords:** Simiao decoction, gouty arthritis, network pharmacology approach, gut inflammation, gut microbiota

## Abstract

Simiao decoction, a classical traditional Chinese medicine (TCM) formula, has been widely used for thousands of years due to its safety and efficiency in treating gouty arthritis. Utilizing serum proinflammatory cytokines and gut ecosystems, this study elucidated the mechanisms of alleviating gouty arthritis by Simiao decoction. Simiao decoction (4.0, 8.0, and 16.0 g/kg) was orally administered to gouty arthritis mice and febuxostat was given as a positive control. The spleen, kidney, and liver indexes indicated that Simiao decoction was safe for the treatment of gouty arthritis in C57BL/6 mice. Besides, our study demonstrated that Simiao decoction was effective for reducing the level of serum uric acid and decreasing MPO, XOD, and ADA activity, as well as alleviating gouty-related symptoms, such as foot swelling and pain. Moreover, Simiao decoction could also reduce some specific serum proinflammatory cytokines including IL-1β, IL-9, IFN-γ, MIP-1α and MIP-1β. We then surveyed the effects of Simiao decoction on the gut ecosystems in a systematic manner by combining network pharmacology, ELISA, western blot, and illumina sequencing. In the murine of model of gouty arthritis, Simiao decoction could suppress NLRP3 inflammasomes expression, reduce gut apoptosis through modulating TNF-α, Caspase 8, and AIFM1 protein expressions, affect lipid metabolism by regulating APOB, LPL, PPARα protein expressions and restore gut microbiota *via* reducing potential pathogens. Overall, these findings suggested that Simiao decoction was an effective therapeutic drug for gouty arthritis and the gut ecosystem might act as a potential anti-inflammatory target of Simiao decoction.

## Introduction

Gout is characterized by recurrent attacks involving inflammatory arthritis, metabolic hyperuricemia and deposition of urate crystals in and around joints (Neogi, 2011). In recent decades, the prevalence of gouty arthritis seems to be increasing worldwide, which is potentially attributable to dietary and lifestyle shift, improved medical care, and increased longevity (Kuo et al., 2015). Most individuals with gouty arthritis suffer musculoskeletal disabilities that impact day-to-day activities, such as self-care, recreation, and work (Stewart et al., 2018). The deposits activate NLR family pyrin domain containing 3 (NLRP3) inflammasome signaling pathways, resulting in the production of inflammatory interleukin-1 beta (IL-1β) (Kingsbury et al., 2011). The most important risk factor for the development of gouty inflammation is hyperuricemia which results from purine metabolism disorder (Torralba et al., 2012). Although extensive knowledge about the pathogenesis of gouty arthritis has been reported, the treatment of gouty arthritis still remains a challenge. Anti-inflammatory prevention is costly and has many contraindications while adherence to urate-lowering therapy is poor (Keenan et al., 2011; Wilson and Saseen, 2016). Therefore, it is imperative to explore new available approaches for the treatment of gouty arthritis, especially complementary and alternative medicine.

Tradition Chinese medicine (TCM) has been widely employed in the treatment of gouty arthritis in China for thousands of years. Recently, in favor of the safety and the advantage of the multitarget in TCM, it has attracted increasing attention in gout treatment (Li et al., 2013). Among numerous effective prescriptions, Simiao decoction, which is composed of *Atractylodes lancea (Thunb.) DC*. (Asteraceae family)*, Phellodendron amurense Rupr*. (Rutaceae family)*, Achyranthes bidentata Blume* (Amaranthaceae family), and *Coix lacryma-jobi* var. *ma-yuen (Rom.Caill.) Stapf* (Poaceae family), is one of the most frequently used prescriptions for gouty arthritis therapy (Liu et al., 2017). Previous studies have shown that Simiao decoction can significantly relieve the symptoms of gouty arthritis through anti-inflammatory effects and urate reducing (Qiu et al., 2008; Hu et al., 2010; Zeng et al., 2014). Nevertheless, the pharmacodynamic mechanism of Simiao decoction remains elusive due to its multitarget characteristics.

Network pharmacology provides novel insights to medical studies, especially for herbal medicine. TCM network pharmacology focuses on the wholeness and systematic interaction between components, targets and diseases (Dong et al., 2019). Although the network, which comprises a large number of compounds and targets, can explain the holistic and complex effects of TCM, numerous mechanistic hypotheses remain to be tested (Huang et al., 2018). To overcome this shortcoming, the current study uncovers the pharmacological mechanisms of Simiao decoction by combining network pharmacology with *in vivo* animal experiments.

Recently, mounting evidence demonstrated that TCM modulation of gut microbiota structure could be one of the mechanisms by which TCM relieves disease (Tong et al., 2018; Yu et al., 2018). When orally administered, TCM ingredients, such as polysaccharides, alkaloids and organic acids, were proven to be prebiotics which could induce changes in the gut microbiota favorable for host health (Xu et al., 2017). The improved gut microbiota could further enhance mucosal immunity and intestinal absorption of the bioactive small molecular chemicals in TCM (Xu et al., 2017). In addition, gut microbiota could biotransform TCM chemicals into metabolites that harbored different bioavailabilities and bioactivities/toxicities in contrast with their precursors (Niu et al., 2013). Accumulating evidence indicated that gut microbiota played a significant role in purine metabolism, urate excretion and NLRP3 inflammasome activation (Chiaro et al., 2017; Zhu et al., 2018; Singh et al., 2019). In gout patients, there were also disorders of the gut microbiome (Guo et al., 2016; Shao et al., 2017). Then, gut microbiota affect the host heath by regulating gut cells. In addition, TCM could be directly absorbed by gut cells to affect the host health. Overall, gut ecosystems, which are comprised of gut microbiota and intestinal wall cells, are vital for TCM to treat gouty arthritis.

The present study was carried out in C57BL/6 mice to assess the therapeutic safety and effectiveness of Simiao decoction. Moreover, comparisons between the target genes of Simiao decoction and gouty arthritis disease-related genes were conducted to uncover potential targets of Simiao decoction for the treatment of gouty arthritis. Furthermore, the potential targets were validated in the colon tissues *in vivo*. Finally, the effects of Simiao decoction on the gut microbiota and serum proinflammatory cytokines were investigated. Notably, this study was beneficial for elucidating the pharmacological mechanisms of Simiao decoction.

## Materials and Methods

### Preparation of Simiao Decoction

Simiao decoction was composed of Atractylodes lancea (Thunb.) DC. (12 g), Phellodendron amurense Rupr. (12 g), Achyranthes bidentata Blume (12 g), and Coix lacryma-jobi var. ma-yuen (Rom.Caill.) Stapf (30 g), which were purchased from Zhejiang Chinese Medical University Medicine Yinpian Factory (Hangzhou, China) and authenticated by Prof. Chengping Wen and Dr. Lin Huang. The voucher specimen (No. 20190128-1 for Atractylodes lancea (Thunb.) DC., No. 20190128-2 for Phellodendron amurense Rupr., No. 20190128-3 for Achyranthes bidentata Blume., No. 20190128-4 for Coix lacryma-jobi var. ma-yuen (Rom.Caill.) Stapf) have been deposited by Zhixing He at the Institute of Basic Research in Clinical Medicine of Zhejiang Chinese Medical University (Hangzhou, China).

To prepare this formula, a total of 66 g of mixed Simiao decoction crude herbs were immersed in 660 ml of distilled water for 1 h and then boiled and decocted for 1 h to yield a final volume 100 ml. The decoction was filtered with eight layers of surgical gauze. Herb residues were again soaked in 300 ml water, boiled for 1 h, and filtered again. Both filtered decoctions were combined and concentrated with rotary evaporation at 55°C until a final volume of 50 ml was reached, then lyophilized with freeze dryer to get the extract. Finally, the dried residue was dissolved in water to obtain Simiao oral solution with three concentrations of 0.2, 0.4, and 0.8 g/ml. Raw materials were stored at room temperature. Simiao preparations were stored at -40°C. For chemical identification of Simiao preparations, UPLC chromatographic analysis was conducted. The UPLC analysis procedure and characteristic chromatogram of Simiao decoction were shown in **Figure S1**.

### Animal Experiments

Forty-two specific pathogen-free (SPF) male C57BL/6 mice (male, 4 weeks old, 15 ± 3 g) were purchased from Shanghai SLAC Laboratory Animal Co., Ltd., and maintained in the SPF environment of the Zhejiang Chinese Medical University laboratory animal research center. All animal experiments were approved by the Ethics Committee of Zhejiang Chinese Medical University with approval number ZSLL-2018-042. All mice were housed under a 12 h/12 h light/dark cycle and constant temperature (25 ± 1°C) and humidity (50 ± 5%) with food and water available ad labium.

After 7 days of acclimatization, all 42 mice were randomly divided into two groups: 1) control group (CT, N=7 mice/group), fed a normal diet, and 2) gouty arthritis model group (N=35 mice/group), fed a high-fat diet (10% yeast extract) and injected with monosodium urate crystal (MSU) crystals (25 mg/ml in PBS/mouse per 10 days). After 7 days, the gouty-arthritis model group was further divided into five subgroups (eight per group): I) model group, oral gavage with sterile water each day; II) low-dose of Simiao group, oral gavage with 4.0 g/kg Simiao decoction each day; III) middle-dose of Simiao group, oral gavage with 8.0 g/kg Simiao decoction each day; IV) high-dose of Simiao group, oral gavage with 16.0 g/kg Simiao decoction each day; V) febuxostat group, oral gavage with 5.0 mg/kg febuxostat each day. Throughout the trial, all mice were weighed each week, and the drug doses were adjusted accordingly.

Dosage of Simiao decoction and febuxostat were determined based on the conversions from clinical adult dosages. The dosage of Simiao decoction for adult is 66 g (the total raw materials)/day, equivalently, the dosage for mice is 8.0 g/kg/day calculated by the formula that converts dosage of human into that of mouse according to the respective body surface areas in accordance with the Chinese Medicine Pharmacology Research Technology. Therefore, this study set 4.0, 8.0, and 16.0 g/kg/day Simiao decoction as the low, middle and high dose in mice, respectively.

At the end of the trial (42 days), samples were collected at 12 h after the last drug administration and injection of MSU. Blood samples were obtained from the eye socket vein of each mouse and centrifuged at 1300 g for 10 min at 4°C. Fecal material was removed from the colon and stored at -80°C for further analysis. Then, the colon, liver and foot joint tissues were immediately isolated, snap-frozen in liquid nitrogen, and stored at -80°C.

### MSU Crystal Preparation and Induction of Foot Joint Inflammation

MSU crystals were prepared by dissolving monosodium urate (800 mg) in boiling milli-Q water (155 ml) that contained NaOH (5 ml). After adjusting the pH to 7.2, the solution was then cooled gradually by stirring at room temperature. The crystals were collected by centrifugation (3000 g, 2 min, and 4°C), evaporated, and sterilized by heating at 180°C for 2 h. MSU crystals were stored in a sterile microtube until use.

Joint inflammation was induced by the intra-articular (i.a.) administration of MSU (1.0 mg/40 μl) into the right foot joint of each mouse under isoflurane anesthesia. Control mice received an intra-articular injection of 40 μl of PBS also into the right hind paw.

Foot swelling was quantified by measuring the thickness of the paw with a caliper (Meinaite, Germany) before stimulus and 48 h after the i.a. administration of MSU. The thickness of the paw was determined for each mouse by the difference between the time point indicated in the figures and the baseline value (zero time point). The edema value is expressed as the ratio of Δ mm/mm (zero time point) of the joint.

Foot pain was quantified by measuring the mechanical withdrawal threshold (MWT) with the von Frey hairs (Stoelting, Wood Dale, IL) using the up and down method as described by Chaplan et al. (1994). In a quiet, temperature-controlled room, mice were individually placed in testing chambers with metal mesh floors at least 30 min before testing for acclimation. Each test began with 0.16 g force delivered perpendicularly to the central plantar surface of either hindpaw for 3 s with a hair. Positive responses included sudden paw withdrawal, flinching, or paw licking, and no response was negative. The threshold force required to elicit withdrawal (50% hind paw withdrawals) was determined for right hind paws (Marcotti et al., 2018). The threshold force data are presented as the difference from baseline values, with a negative value indicating mechanical allodynia. Threshold values were assessed in mice at 48 h after injection of MSU or vehicle (PBS) solutions in the right hind paw. All behavioral tests were performed by an investigator (Mingzhu Wang) who was blinded to the experimental design.

### Cytokine and Enzyme Measurement

A volume of 25 μl of serum was taken for the determination of cytokine and chemokine levels using a commercial multiplex mouse cytokine magnetic bead-based immunoassay (Bio-Plex Pro Mouse Cytokine 23-plex Assay, Bio-Rad Laboratories) according to the manufacturer's instructions. The mean fluorescence intensity from all the bead combinations tested was analyzed using the Bio-Plex system equipped with Bio-Plex Manager Software v6.0 (Bio-Rad Laboratories).

The analysis of cytokines in the colon was evaluated. Colons were homogenized in a high-speed homogenizer with buffer solution (1 ml/0.05 g) and centrifuged for 10 min at 12000 rpm at 4°C. The supernatants collected were used to detect cytokines (sIgA, TNF-α, IL-6, and IL-1β) by mouse ELISA using commercial kits (CUSABIO, Wuhan, China) according to the manufacturer's instructions. The minimum detection limits were 15.6 ng/ml for sIgA and TNF-α 1.56 pg/ml for IL-6, and 6.25 pg/ml for IL-1β. The concentration of protein in the supernatant was measured by a micro bicinchoninic acid (BCA) method. The levels of cytokines were expressed in ng or pg (cytokines) per mg (total protein).

Upon thawing the foot joint or liver samples, the tissues (0.05 g of tissue per 1.0 ml buffer solution) were homogenized and centrifuged for 10 min at 12,000 rpm at 4°C. The supernatants were evaluated for myeloperoxidase (MPO) activity in foot joint tissues and xanthione oxidase (XOD) and adenosine deaminase (ADA) activity in liver tissues, in accordance with the manufacturer's instructions (Jiancheng, Nanjing, China). The results are expressed in U/mg (total protein). In addition, the serum uric acid (UA), creatine (Cr) and alanine transaminase (ALT) levels were measured *via* an enzymatic-colorimetric method, using standard test kits on a TBA-40FR automated biochemical analyzer (Toshiba, Tokyo, Japan).

### Predicated Targets Analysis

The compounds of the four herbs in Simiao decoction and their targeted genes were downloaded from the Traditional Chinese Medicine Integrated Database (TCMID, http://www.megabionet.org/tcmid/) (Huang et al., 2017) for further analysis (Huang et al., 2016). Fifteen compounds in Simiao decoction were enrolled in this study, including adeninenucleoside, 2,6-ditertbutyl-4methyl phenol, atractyloside, β-elemene, atractyloside, α-eudesmol, obaculactone, β-sitosterol, obakulactone, dibutyl phthalate, chrysophanol, octadecenoic acid, coixan, rhamnose, and galactose. The related targets were filtered by the recommended confidence range defined by STITCH (low confidence: scores < 0.4; medium: 0.4 to 0.7; high: > 0.7). Genes or proteins related to gout arthritis therapy were identified from Drugbank (https://www.drugbank.ca/). Protein-protein interactions collected from Biogrid (https://thebiogrid.org) were used to construct the gene association network. The network was generated and visualized by Cytoscape 3.4.0.

### Gut Microbiota Analysis

Total genomic DNA was extracted from each stool sample using a stool DNA isolation kit (Tiangen, Biotech Co., Ltd., Beijing, China) according to the manufacturer's protocols. Qualified DNA was amplified with broad-range bacterial primers (319F: 5'-ACTCCTACGGGAGGCAGCAG-3' and 806R: 5'-GGACTACHV GGGTWTCTAAT-3') targeting the V3-V4 region of the 16S rRNA gene. The volume of the PCR reaction was 30 μl with 15 μl of Phusion High-Fidelity PCR Master Mix with HF Buffer (NEB), 0.2 μM forward and reverse primers, and 10 ng of template DNA. The reactions were hot-started at 98°C for 30 s, followed by 30 cycles of 98°C for 15 s, 58°C for 15 s, and 72°C for 15 s, with a final extension step at 72°C for 1 min. Subsequently, the amplicons were purified according to standard procedures, quantified, pooled and sequenced with the MiSeq Reagents Kit v3 (600 cycles, Illumina) according to the manufacturer's instructions. The sequencing reaction was conducted by Hangzhou Legenomics Bio-Pharm Technology Co., Ltd., Zhejiang, China.

Sequences were analyzed using Quantitative Insights into Microbial Ecology (QIIME) (Caporaso et al., 2010). High-quality reads were selected and all of the effective reads were clustered into operational taxonomical units (OTUs). The reads with > 97% similarity were assigned to the same OTUs by UCLUST (Edgar, 2010). Bacterial taxonomy was assigned by using the SILVA (Quast et al., 2013) and NCBI databases (Coordinators, 2018). The OTU table was imported into R software and alpha and beta diversity metrics were computed using the “vegan” package. To analyze the alpha diversity, Shannon and Chao1 indices were performed by using R software. For the beta diversity analysis, the principal coordinate analysis (PCoA) based on the unweighted UniFrac distance matrices were visualized by R software. OTUs with >0.05% mean abundance in one sample and observed in >10% of the samples were included in differential analyses. To reveal differences in deeper data of microbial diversity between the samples, significance test was conducted with the linear discriminant analysis (LDA) effect size (LEfSe) method (Segata et al., 2011), with a set logarithmic LDA score of 2.0. Additionally, Spearman's rank correlation was conducted between gut inflammatory cytokines and altered microbial genera that were found to be significantly different.

### Western Blot Analysis

Colon samples were homogenized with 10 equivalent volumes of RIPA lysis buffer supplemented with 1 mM PMSF (a protease inhibitor) in an ice bath for 30 min and then centrifuged (12,000×g, 10 min) to extract total proteins. The protein concentration was determined by using a BCA protein assay kit (Beyotime Biotechnology, Shanghai, China). Cell lysates (40 μg) were separated on a 7.5/10/15% SDS-PAGE gel and then transferred onto a nitrocellulose membrane (Pall Corporation, MI, USA). The nonspecific binding sites of the membranes were blocked with 5% BSA in TBST (Tris-buffered saline with 0.1% Tween-20) for 1 h at room temperature and incubated with primary antibody overnight at 4°C. The membranes were washed with TBST and incubated with the appropriate secondary antibody for 1 h. The Western blot results were scanned and validated with an infrared imaging system (Odyssey CLx; LI-COR Biosciences). Gel quantification was performed using ImageJ software.

The following antibodies were obtained from commercial sources as indicated: NLRP3 (1:1000 dilution; Cell Signaling Technology, #15101); IL-1β (1:1000 dilution; Cell Signaling Technology, #12426); Caspase 1(1:1000 dilution; Abcam, #ab179515); ASC(1:1000 dilution; Cell Signaling Technology, #67824); Phospho-Stat3 (1:2000 dilution; Cell Signaling Technology, #9145); APOB (1:10000 dilution; Abcam, #ab157205); PPARα (1:1000 dilution; Invitrogen, # PA1-822A); Caspase 8 (1:1000 dilution; Cell Signaling Technology, #4790); Fibronectin (1:10000, Abcam, #ab45688); AIFM1 (1:1000 dilution; Abcam, #ab 32516); c-FOS (1:1000 dilution; Cell Signaling Technology, #2250); c-Jun (1:1000 dilution; Cell Signaling Technology,#9165); β-actin(1:5000; R&D system, #MAB8929); Dylight 800, Goat anti-Rabbit IgG (1:10000 dilution; Immunoway, #RS23920); and Dylight 680, Goat anti-Mouse IgG (1:10000 dilution; Immunoway, #RS23710).

### Statistical Analysis

Statistical analyses were performed using SPSS software version 16.0. Based on the normality test and homogeneity of variance, one-way ANOVA or the rank sum test was adopted for data analysis. In addition, *p* values were adjusted using Benjamini-Hochberg method to control the false discovery rate (FDR) and an adjusted *p*-value <0.05 was considered statistically significant.

## Results

### Safety of Simiao Decoction

**Figure 1:**
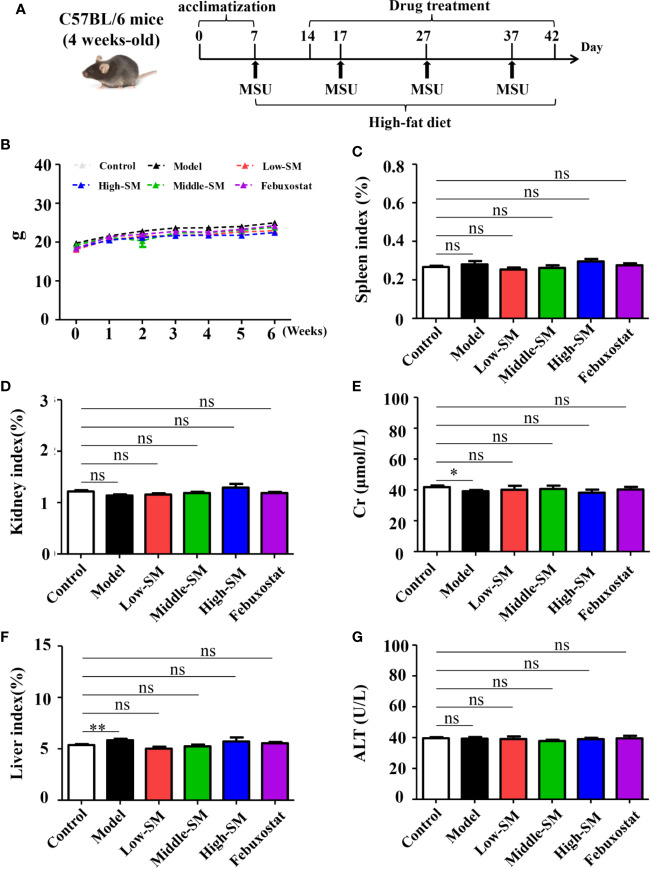
Safety of Simiao decoction in the treatment of gouty arthritis mice. A diagram of the experimental treatments is shown **(A)**. Effects of Simiao decoction on body weight **(B)**, spleen index **(C)**, kidney index **(D)**, creatine **(E)**, liver index **(F)**, and alanine aminotransferase **(G)**. “ns” represents not significant; “*” represents *p* < 0.05; and “**” represents *p* < 0.01. Cr, creatine; ALT, alanine aminotransferase. N = 7/group.

All mice in the six groups had normal breathing without abnormal behaviors or automatic behaviors. No mortality occurred throughout the experiment. The weight gains of the mice in all six groups were consistent (**Figure 1B**). At the end of the experiment, no significant differences was found between the control group and the three Simiao-treated groups with respect to spleen, kidney and liver indices or Cr and ALT concentrations (**Figure 1**). In addition, the mice in the experimental group had lower Cr concentrations and higher liver indices compared with control group (**Figures 1E, F**).

The above results indicated the safety of Simiao decoction on male C57BL/6 mice.

### Simiao Decoction Attenuated Gouty Symptoms

**Figure 2:**
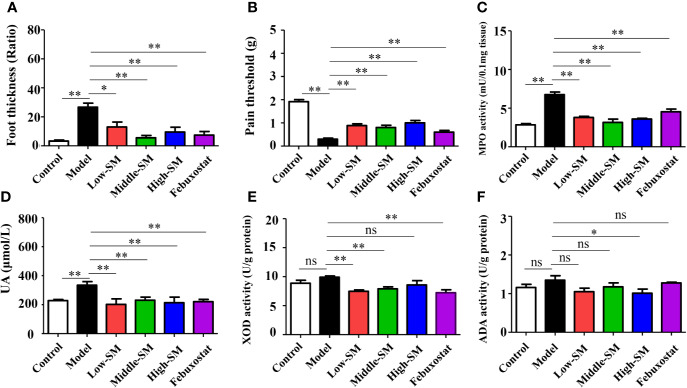
Therapeutic efficacy of Simiao decoction in the treatment of gouty arthritis mice. Effects of Simiao decoction on foot thickness **(A)**, pain threshold **(B)** of paw, MPO activity **(C)** in foot tissue, UA concentration in serum **(D)**, XOD activity **(E)**, and ADA activity **(F)** in liver tissue. “ns” represents not significant; “*” represents *p* < 0.05; and “**” represents *p* < 0.01. MPO, myeloperoxidase; UA, uric acid; XOD, xanthione oxidase; ADA, adenosine deaminase. N = 7/group.

After being fed a high-fat diet and injected with MSU crystals, the mice showed higher levels of uric acid MPO activity, accompanying gouty symptoms, including foot swelling, low pain threshold (**Figure 2**). Both Simiao decoction and febuxostat significantly attenuated gouty symptoms in murine model with gouty arthritis (**Figure 2**). Simiao decoction had similar therapeutic effects among the different doses for the treatment of gouty arthritis. To further clarify the mechanism by which uric acid was reduced, both XOD and ADA activities were determined. There were no significant differences in both XOD and ADA activities between the control and experimental groups (**Figures 2E, F**). As an inhibitor of XOD, febuxostat could significantly block XOD activity in the mice with gouty arthritis, but no effect was observed on ADA activity (**Figures 2E, F**). Low- and middle-dose Simiao decoction reduced uric acid by inhibiting XOD activity, while ADA activity was blocked by high-dose Simiao decoction.

These results showed that oral administration of Simiao decoction alleviated the gouty symptoms that induced by the high-fat diet and injection of MSU crystals.

### Effects of Simiao Decoction on Proinflammatory Cytokines

**Figure 3:**
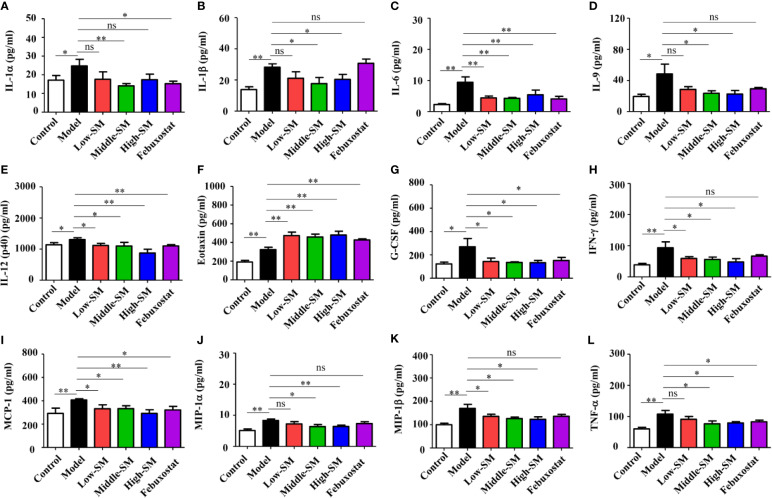
Effects of Simiao decoction on serum proinflammatory cytokines (N = 7/group). IL-1α **(A)**, IL-1β **(B)**, IL-6 **(C)**, IL-9 **(D)**, IL-12(p40) **(E)**, eotaxin **(F)**, G-CSF **(G)**, IFN-γ **(H)**, MCP-1 **(I)**, MIP-1α **(J)**, MIP-1β **(K)**, and TNF-α **(L)**. “ns” represents not significant; “*” represents *p* < 0.05; and “**” represents *p* < 0.01.

The levels of 23 proinflammatory cytokines in serum were investigated in the six groups of C57BL/6 mice using the Bio-Plex Pro mouse cytokine GI 23 plex panel. No significant difference in IL-3 was observed among the six groups (**Figure S2**), for the other 22 proinflammatory cytokines, the gouty arthritis mice showed a significantly increased level compared to that of the control mice. Neither Simiao decoction nor febuxostat had any effects on several proinflammatory cytokines, including IL-2, IL-4, IL-5, IL-12(p70), IL-10, IL-13, IL-17, KC, GM-CSF, and RANTES (**Figure S2**). Conversely, IL-1α, IL-6, IL-12(p40), G-CSF, MCP-1, and TNF-α, which could be induced by gouty arthritis, were significantly reduced by Simiao decoction and febuxostat (**Figure 3**). The production of eotaxin was significantly increased by Simiao decoction and febuxostat. There were some other proinflammatory cytokines that were significantly reduced by Simiao decoction but were not affected by febuxostat, including IL-1β, IL-9, IFN-γ, MIP-1α and MIP-1β **Figure 3**).

These results clearly showed that the reduction in proinflammatory cytokines was one of the mechanisms by which Simiao decoction ameliorated gouty arthritis.

### Validation of Potential Pharmacological Targets of Simiao Decoction in Alleviating Gouty Arthritis

To further explore the underlying pharmacological mechanisms of Simiao decoction in gouty arthritis and find hub proteins for disease intervention, PPI analysis were carried out to explore the Simiao decoction targets and gouty arthritis related proteins. We found that 17 formula targets overlapped with gouty arthritis related-genes, including *STAT3*, *FN1*, *APOB*, *AIFM1*, *PTGER1*, *ALB*, *TNF*, *VEGFA*, *LPL*, *PPARa*, *SREBF2*, *CASP8*, *JUN*, *FOS*, *AR*, *TTR*, and *AKR1B1* (**Figure S3**).

**Figure 4:**
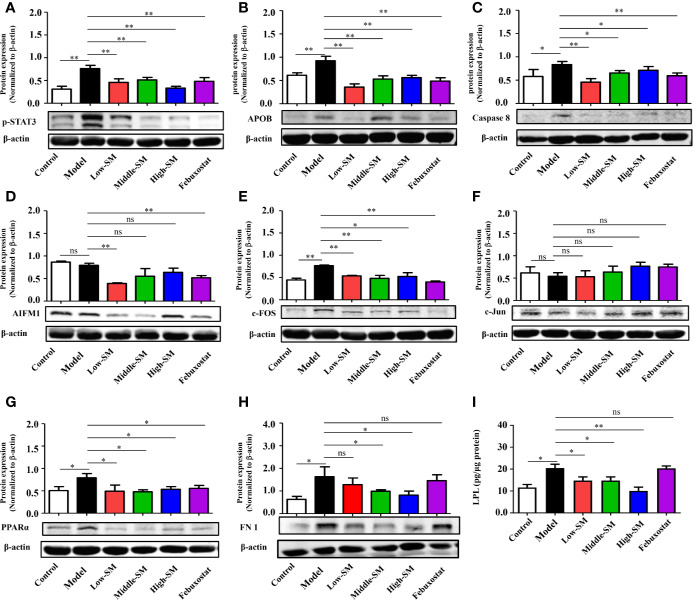
Validation of potential targets regulated by Simiao decoction in gouty arthritis mice. Protein expression of STAT3 **(A)**, APOB **(B)**, caspase 8 **(C)**, AIFM1 **(D)**, FOS **(E)**, Jun **(F)**, PPARα **(G)**, FN1 **(H)**, and LPL **(I)** in colon tissue (N = 3/group). “ns” represents not significant; “*” represents *p* < 0.05; and “**” represents *p* < 0.01. STAT3, signal transducer and activator of transcription 3; APOB, apolipoprotein B; AIFM1, apoptosis-inducing factor, mitochondrion-associated, 1; PPARα, peroxisome proliferator-activated receptor alpha; FN1, fibronectin 1; LPL, lipoprotein lipase.

Simiao decoction was absorbed by the host in the intestine after oral gavage. Hence, to further validate the overlap between Simiao decoction targets and gouty arthritis related-genes, nine targets were selected according to the expression in colon tissues. The expression levels of nine proteins in colon tissues were detected by western blotting and ELISA. As shown in **Figure 4**, the protein expression levels of p-STAT3, APOB, CASP8, c-FOS, PPARα FN1, and LPL were markedly increased in the experimental group compared with those of the control group. Simiao decoction notably reduced the expression levels of the aforementioned seven proteins. Febuxostat reduced the expression levels of p-STAT3, APOB, CASP8, c-FOS, and PPARα. In contrast, there were no significant differences in AIFM1 and c-Jun protein expression between the experimental and control groups.

The above data together indicated that Simiao decoction exerted significant effects on some targets predicted by a network pharmacology approach. The therapeutic targets through which Simiao decoction alleviated gouty arthritis were p-STAT3, APOB, CASP8, c-FOS, PPARα, FN1, and LPL.

### Simiao Decoction Reduced Gut Inflammation by Inhibiting NLRP3 Inflammasomes

**Figure 5:**
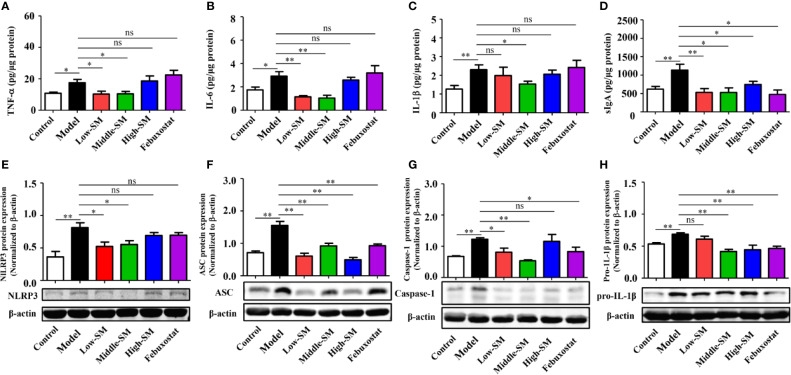
Reduction in gut inflammation by Simiao decoction in the treatment of gouty arthritis mice. Effects of Simiao decoction on TNF-α **(A)**, IL-6 **(B)**, IL-1β **(C)** and sIgA **(D)** concentrations. Protein expression of NLRP3 **(E)**, ASC **(F)**, caspase 1 **(G)**, and pro-IL-1β **(H)** in colon tissue (N=3/group). “ns” represents not significant; “*” represents *p* < 0.05; and “**” represents *p* < 0.01.

Increasing evidence has demonstrated that gut inflammation can modulate innate and adaptive immune responses and impact the pathogenesis of inflammatory diseases. In the present study, gut inflammation in the mice with gouty arthritis was tested. As shown in **Figure 5**, the concentrations of TNF-α, IL-6, IL-1β and sIgA in colon tissue (N=7/group) were notably increased in the experimental model of gouty arthritis compared with those of the normal control mice. At a concentration of 8.0 g/kg (middle dose), Simiao decoction significantly reduced the levels of these four cytokines. Meanwhile, febuxostat did not have any noticeable effects on TNF-α, IL-6, and IL-1β but decreased the level of sIgA.

Colon samples (N=3/group) were collected to investigate the expression of the NLRP3 inflammasome. In the colon, NLRP3 inflammasome was activated in the mice model of gout arthritis (**Figures 5E–H** ), while production of NLRP3, ASC, and Caspase-1 were reduced by Simiao decoction and febuxostat.

Oral administration of Simiao decoction diminished the inflammatory cytokine levels and inhibited NLRP3 inflammatory activation in colon tissue, demonstrating the suppressive effects of Simiao decoction on gut inflammation.

### Simiao Decoction Altered the Composition and Function of Gut Microbiota

**Figure 6:**
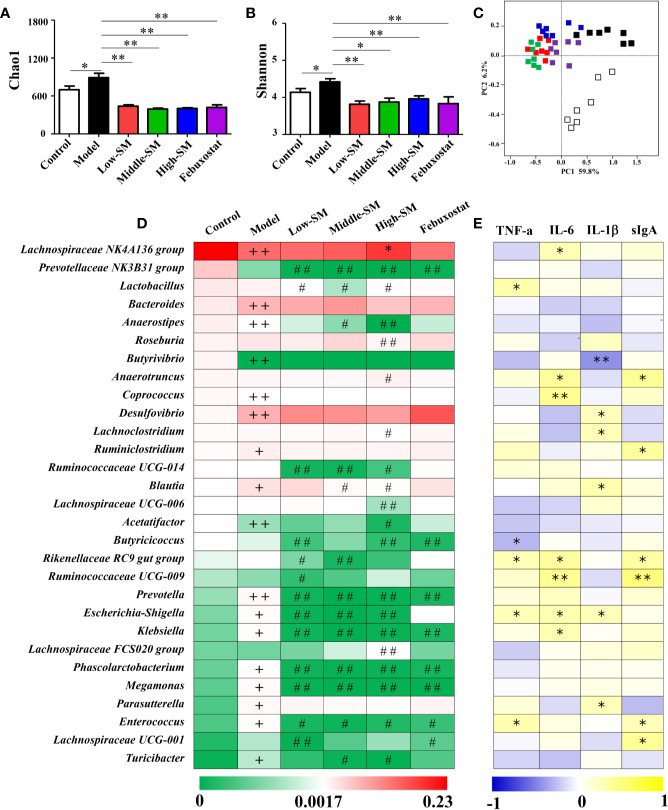
Alterations in gut microbiota caused by Simiao decoction in the treatment of gouty arthritis mice. Effects of Simiao decoction on Chao1 **(A)** and Shannon **(B)** indices. PCoA score plots of gut microbiota based on UniFrac distance matrices **(C)**. The abundances of 30 altered genera are shown as heatmaps **(D)**. Spearman's correlations between 30 altered genera and gut inflammatory cytokines are shown as heatmaps **(E)**. “+” represents *p* < 0.05 in the comparison with control group; “+ +” represents *p* < 0.01 in the comparison with control group; “#” represents *p* < 0.05 in the comparison with model group; “# #” represents *p* < 0.01 in the comparison with model group; “*” represents the Spearman rho value <0.05; and “**” the Spearman rho value *p* < 0.01. N = 7/group.

Alterations in gut inflammation have emerged as a factor that influences gut microbiota. To examine whether the alleviation of gouty arthritis effects by Simiao decoction were associated with gut microbiota, the bacterial 16S rRNA V3-V4 region in colon feces were sequenced. The alpha diversity indices for the Chao1 and Shannon index were significantly different among the various groups (**Figures 6A, B**). There was a positive association between alpha diversity indices and the severity of gouty arthritis in the mice with gouty arthritis. Beta diversity was assessed by PCoA on weighted UniFrac distance matrices. Simiao decoction significantly influenced the gut microbiota composition and separated the control model and Simiao decoction-treated model mice by PCoA (**Figure 6C**). The gouty arthritis model also induced separation in the gut microbiota composition of the experimental and control group (**Figure 6C**). There was no significant separation in the gut microbiota composition among the three concentrations of Simiao decoction-treated groups (**Figure 6C**).

The heatmap of 30 altered genera in five groups was shown in **Figure 6D**. The dominant three genera in the control group were the *Lachnospiraceae* NK4A136 group, *Prevotellaceae* NK3B31 group and *Lactobacillus*. The dominant three genera in the other four groups were *Lachnospiraceae* NK4A136 group, *Bacteroides* and *Desulfovibrio*. Additionally, genera with significant differences among different groups were demonstrated using LEfSe analysis (**Figure S4**). In comparison with the control group, 13 genera (*Desulfovibrio*, *Bacteroides*, *Bilophila*, *Blautia*, *Ruminiclostridium*, *Parasutterella*, *Turicibacter*, *Prevotella*, *Megamonas*, *Enterococcus*, *Escherichia-Shigella*, *Klebsiella*, and *Phascolarctobacterium*) significantly increased and 5 genera (*Lachnospiraceae* NK4A136 group, *Anaerostipes*, *Butyrivibrio*, *Acetatifactor*, and *Coprococcus*) significantly decreased in murine of model gouty arthritis. Furthermore, in contrast with the control model, the populations of potential pathogens were significantly downregulated in the experimental model treated with three concentrations of Simiao decoction, including *Prevotella*, *Escherichia-Shigella*, *Klebsiella*, *Megamonas*, *Enterococcus*, and *Phascolarctobacterium*. Additionally, *Prevotellaceae* Nk3b31 and *Ruminococcaceae* UCG-014 were also significantly downregulated in murine model of gouty arthritis by Simiao decoction.

Spearman's rank correlation method was conducted to further elucidate the association between the 30 altered genera and gut inflammation. As shown in **Figure 6E**, statistically significant negative correlations between *Butyrivibrio* and IL-1β, *Butyricicoccus* and TNF-α were identified. There were significant positive correlations among the following factors: *Lachnospiraceae* NK4A136 group, *Coprococcus*, *Klebsiella* and IL-6; *Lactobacillus* and TNF-α; *Desulfovibrio*, *Lachnoclostridium*, *Blautia*, *Parasutterella*, and IL-1β; and *Ruminiclostridium*, *Lachnospiraceae* UCG-001, and sIgA. Remarkably, some genera were positively correlated with at least two gut inflammatory cytokines including *Anaerotruncus*, *Rikenellaceae* RC9 gut group, *Ruminococcaceae* UCG-009, *Escherichia-Shigella* and *Enterococcus*.

Collectively, these results indicated that gut microbiota responded to gouty arthritis and Simiao decoction, and Simiao decoction could alleviate gouty arthritis by altering gut microbial composition.

## Discussion

**Figure 7:**
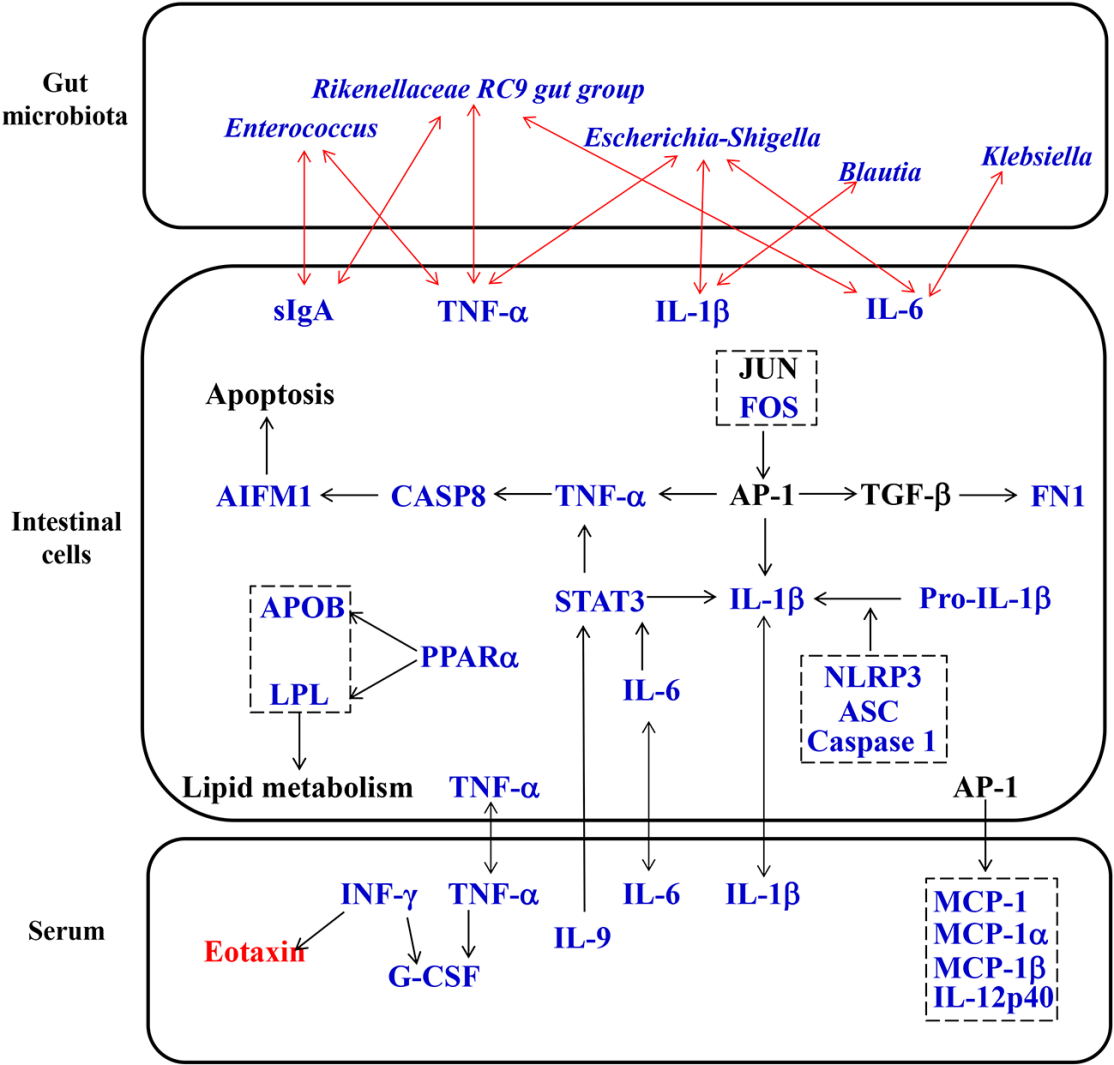
Proposed pharmacological mechanisms of Simiao decoction in the treatment of gouty arthritis. Red text represents the increase caused by Simiao decoction; blue text represents the decrease caused by Simiao decoction.

Many randomized controlled trials have proven that Simiao decoction is an effective therapeutic medicine for gouty arthritis through anti-inflammatory and urate reducing effects (Qiu et al., 2008; Zeng et al., 2014; Liu et al., 2017). Although previous reports have shown that Simiao decoction enhances urate excretion and reduce serum urate levels by regulating the abnormal expression of renal mURAT1, mGLUT9, and mOAT1 (Hu et al., 2010; Wang et al., 2010), the exact mechanism of its anti-inflammatory effects on gouty arthritis is unclear. In our study, Simiao decoction could alleviate gouty arthritis symptoms in mice with no obvious side effects during the 42 days. Moreover, we also observed that Simiao decoction reduced XOD activity, serum proinflammatory cytokines (IL-1β, IL-9, IFN-γ, MIP-1α and MIP-1β), gut inflammation (NLRP3 inflammasomes), apoptosis (TNF-α, Caspase 8 and AIFM1), and lipid metabolism (APOB, LPL, and PPARα), and inhibited pathogenic gut bacteria (*Klebsiella*, *Blautia*, *Escherichia-Shigella* and *Enterococcus*) in the murine model with gouty arthritis (**Figure 7**).

After oral administration, the gut microbiota profiles can be modulated by ingested TCM compounds. Recently, gut microbiota is identified as an important target of TCM to exert its pharmacological effects (Feng et al., 2019). Analysis of the gut microbiota demonstrated that Simiao decoction improved gut microbiota dysbiosis associated with gouty arthritis. The alterations in gut microbiota caused by Simiao decoction were beneficial for relieving inflammation. In murine model of gouty arthritis, Simiao decoction significantly reduced the abundance of *Prevotella*, which was related with the chronic inflammatory conditions (Larsen, 2017). Moreover, some pathogenic bacteria which positively associated with gut inflammatory cytokines were also reduced by Simiao decoction, including *Klebsiella*, *Blautia*, *Escherichia-Shigella*, and *Enterococcus*. *Klebsiella* is resistant to multiple antibiotics, tend to colonize when the intestinal microbiota is dysbiotic, and elicit severe gut inflammation in the context of a genetically susceptible host (Atarashi et al., 2017). A high *Blautia* abundance together with a high-fat diet was associated with poor clinical prognosis in humans (Luu et al., 2017; Liu et al., 2019). A positive correlation was also observed among the proinflammatory cytokines IL-1β, NLRP3, and CXCL2 with the abundance of the inflammatory bacteria of the taxon *Escherichia-Shigella* (Cattaneo et al., 2017). Intestinal *Enterococcus* could subvert the host immune response and cause infectious disease (Kao and Kline, 2019). Taken together, these results clearly demonstrated that the modulation of gut microbiota was one of the mechanisms by which Simiao decoction alleviated gouty arthritis.

The interactions between TCM and gut microbiota not only lead to changes in gut microbiota composition but also cause the transformation of TCM (Xu et al., 2017). The metabolism of intestinal cells can be influenced by changes in gut microbiota and TCM compounds. It is worth noting that we reported for the first time that gut inflammation was upregulated in the gouty arthritis model. Simiao decoction could reduce gut inflammatory cytokines through inhibiting the NLRP3 inflammasome which was important for gout-induced inflammation (Kingsbury et al., 2011). Moreover, other pharmacological targets of Simiao decoction predicted by the network pharmacology approach were validated *in vivo*. For example, Simiao decoction could reduce STAT3, a component of the IL-6-activated acute phase response factor (Fielding et al., 2008) that was activated in the pathological status, to a nearly normal level. STAT3 inhibition could result in the suppressed TNF-α and IL-1β production in response to MSU crystals (Hall et al., 2018). Besides, the transcription factor AP-1 also reduced production of TNF-α and IL-1β (Hsiang et al., 2005; Bashir et al., 2009) The expression of c-FOS, one component of AP-1, was decreased by Simiao decoction. Moreover, the reduction of the three predicated targets (TNF-α, Caspase 8 and AIFM1) are part of the apoptosis pathway (Liu et al., 2018; Munoz-Pinedo and Lopez-Rivas, 2018; Parker et al., 2019), indicated that the apoptosis pathway was partly inhibited. This study also revealed that Simiao decoction could reduce lipid metabolism *via* APOB, LPL and PPARα for the treatment of gout arthritis. Both apoptosis and lipid metabolism pathways serve pivotal roles in gouty arthritis (Lhotta et al., 1998; Zhang et al., 2019). Overall, the suppressions of gut inflammation, apoptosis and lipid metabolism were the pharmacological mechanisms by which Simiao decoction ameliorated gouty arthritis.

An increase in 22 proinflammatory cytokines was observed in the murine model of gouty arthritis in comparison with that of the control mice. The results of most cytokines were in accordance with clinical trials reported before (Andres et al., 2015; So and Martinon, 2017). The analysis of the proinflammatory cytokines demonstrated that the combination of a high-fat diet and injection of MSU can induce gouty arthritis in mice effectively. Both Simiao decoction and febuxostat reduced some proinflammatory cytokines, including IL-6, IL-12(p40), exotoxin, G-CSF, MCP-1, and TNF-α. However, some proinflammatory cytokines were only reduced by Simiao decoction, including IL-1β, IL-9, IFN-γ, MIP-1α and MIP-1β. These results illustrated that the anti-inflammatory activity of Simiao decoction was better than febuxostat. Moreover, the proinflammatory cytokines affected by Simiao decoction provided useful information for better understanding the pharmacological mechanisms of Simiao decoction and further strengthened the therapeutic values of Simiao decoction.

## Conclusions

The present study focused on the potential effect of Simiao decoction on gouty arthritis. Our results demonstrated that Simiao decoction could alleviate gouty arthritis comprehensively through the modulation of proinflammatory cytokines, gut microbiota, inflammation, apoptosis and lipid metabolism pathways. Our study provides important evidence for developing Simiao decoction as a potential option for treating gouty arthritis and the gut ecosystem may act as a potential area for anti-inflammatory targeting of Simiao decoction. In future, the mechanisms of Simiao decoction revealed by this study are needed to be further investigated in clinical samples.

## Data Availability Statement

The raw sequences of Miseq sequences from 42 mice have been submitted to NCBI Project under accession number PRJNA557078 with NCBI Sequence Read Archive under accession number SRP216637.

## Ethics Statement

The animal study was reviewed and approved by Ethics Committee of Zhejiang Chinese Medical University.

## Author Contributions

ZH and CW conceived of and proposed the idea, designed the study. XL, TS, XW, and MW preformed the experiment. ZH, TS, and LH participated in data analysis. XL and ZH contributed to writing assistance and reading the manuscript. All authors contributed to the article and approved the submitted version.

## Funding

This work was supported by the National Natural Science Foundation of China (grant number 81873269 and 81873145), National Key R&D Program of China (2018YFC1705500).

## Conflict of Interest

The authors declare that the research was conducted in the absence of any commercial or financial relationships that could be construed as a potential conflict of interest.

## Abbreviations

TCM, traditional Chinese medicine; MSU, monosodium urate; MPO, myeloperoxidase; XOD, xanthione oxidase; ADA, adenosine deaminase; UA, uric acid; Cr, creatine; ALT, alanine transaminase; IL, interleukin; G-CSF, granulocyte colony stimulating factor; GM-CSF, granulocyte/macrophage colony stimulating factor; IFN-α, interferon-γ; KC, keratinocyte derived cytokines; MCP-1, monocyte chemotactic protein 1; TNF-α, tumor necrosis factor-alpha; STAT3, signal transducer and activator of transcription 3; FN1, fibronectin1; APOB, apolipoprotein B; AIFM1, apoptosis-inducing factor, mitochondrion-associated 1; PTGER1, prostaglandin E Receptor EP1; ALB, albumin; VEGFA, vascular endothelial growth factor A; LPL, lipoprotein lipase; PPARa, peroxisome proliferator activated receptor alpha; SREBF2, sterol regulatory element-binding protein; CASP8, caspases 8; JUN, one subunit of activator protien-1; FOS, one subunit of activator protien-1; AR, androgen receptor; TTR, transthyretin; AKR1B1, aldo-keto reductase family 1, member B1; sIgA, secretory immunoglobulin A; NLRP3, NLR family pyrin domain containing 3; ASC, apoptosis-associated speck-like protein containing CARD; OTUs, operational taxonomical units; PCoA, principal coordinate analysis; LDA, linear discriminant analysis; QIIME, Quantitative Insights into Microbial Ecology.
