# Supplementary material for: Simiao Decoction Alleviates Gouty Arthritis by Modulating Proinflammatory Cytokines and the Gut Ecosystem
Source: Front Pharmacol. 2020 Jun 24;11:955. doi: 10.3389/fphar.2020.00955 (PMC7327538; doi:10.3389/fphar.2020.00955)
Supplement: Supplementary file 1 [file DataSheet_1.pdf]

Simiao decoction alleviates gouty arthritis by modulating proinflammatory cytokines and the gut ecosystems

Xiaoying Lin#, Tiejuan Shao#, Lin Huang, Xianghui Wen, Mingzhu Wang, Chengping Wen* and Zhixing He*

Institute of Basic Research in Clinical Medicine, College of Basic Medical Science, Zhejiang Chinese Medical University, Hangzhou 310053, China.

***Corresponding author.**
Address: Institute of Basic Research in Clinical Medicine, College of Basic Medical Science, Zhejiang Chinese Medical University, Hangzhou 310053, China.
"#": these authors contributed equally to this work.
Tel.: 086-571-86613587.
E-mail address: wengcp@yeah.net (Chengping Wen), hzx452871069@yeah.net (Zhixing He)

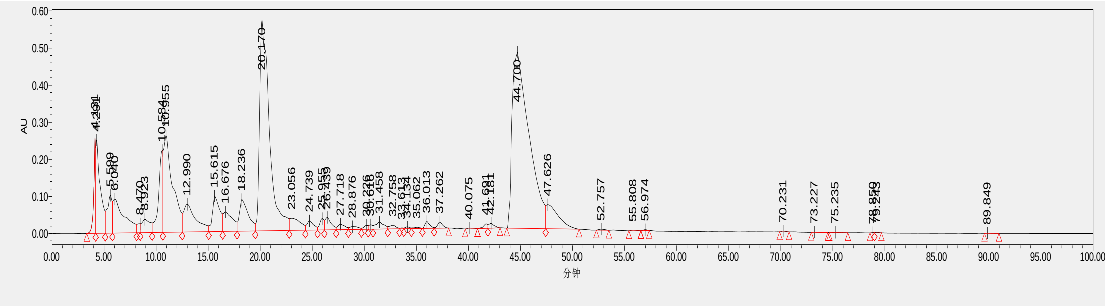

Fig S1 UPLC characteristic chromatogram of Simiao decoction. The UPLC analysis procedure is as follows: Simiao preparations (0.5 g) was added to 10 mL centrifuge tube containing 5.0 mL methanol, sonicated for 45 min at 35 kHz and 25 oC, and then centrifuged at 3500 rpm for 5 min. After filtration with a 0.22 μm filter, the supernatant (1000 μL) was transferred to a 1.5 mL sample vial. The UPLC analysis was carried out on a Waters UPLC (Waters e2695 and a 2998 PDA detector). The chromatographic separation was performed using a Phenomenex Jupiter C18 column (00G-4053-E0) at 25 oC, and 1.5 % aqueous glacial acetic acid (A) and methanol (B) were used as the mobile phase for analysis. The flowrate was set at 0.8 mL /min. The elution conditions were applied with a gradient program as follows: 90 % A–10 % B for 0–10 min, 55 % A–45 % B for 10–60 min, 25 % A–75 % B for 60–85 min, 15 % A–85 % B for 85–95 min, 90 % A–10 % B for 95–100 min. Ten microliters of sample was injected into the UPLC system for analysis and detected at 280-305 nm.

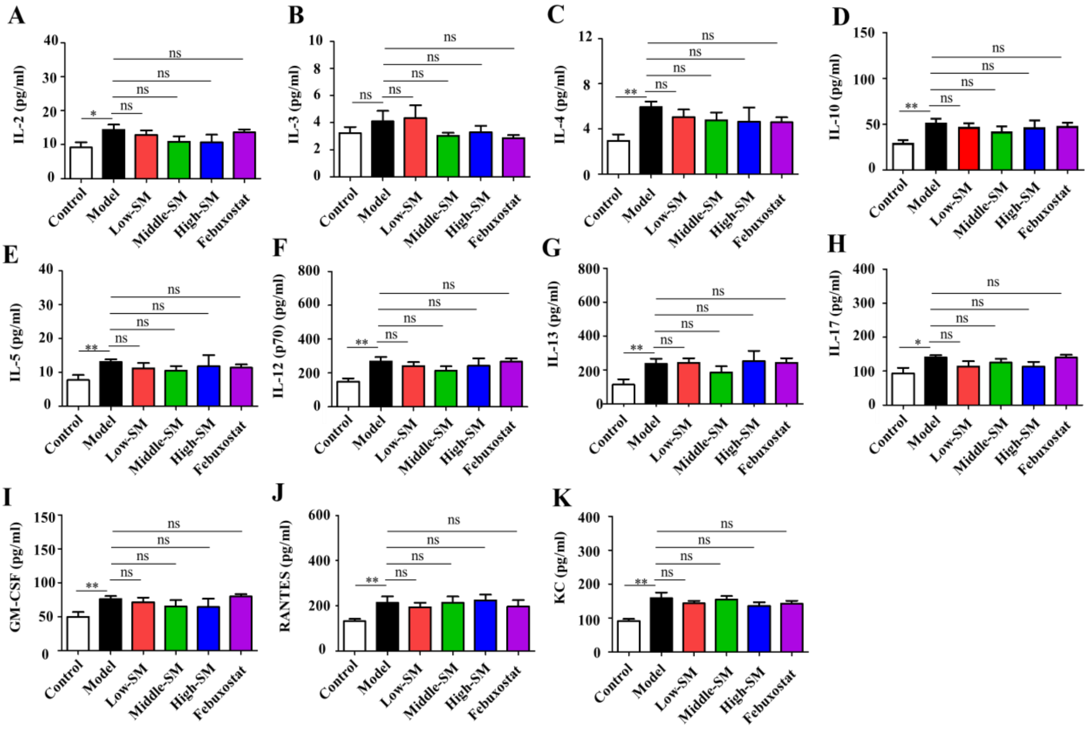

Fig S2 Effects of Simiao decoction on serum proinflammatory cytokines (N = 7/group). IL-2 (A); IL-3 (B); IL-4 (C); IL-10 (D); IL-5 (E); IL-12(p70) (F); IL-13 (G); IL-17 (H); GM-CSF (I); RANTES (J); KC (K). "ns" represents not significant; "*" represents $p<0.05$; and "**" represents $p<0.01$.

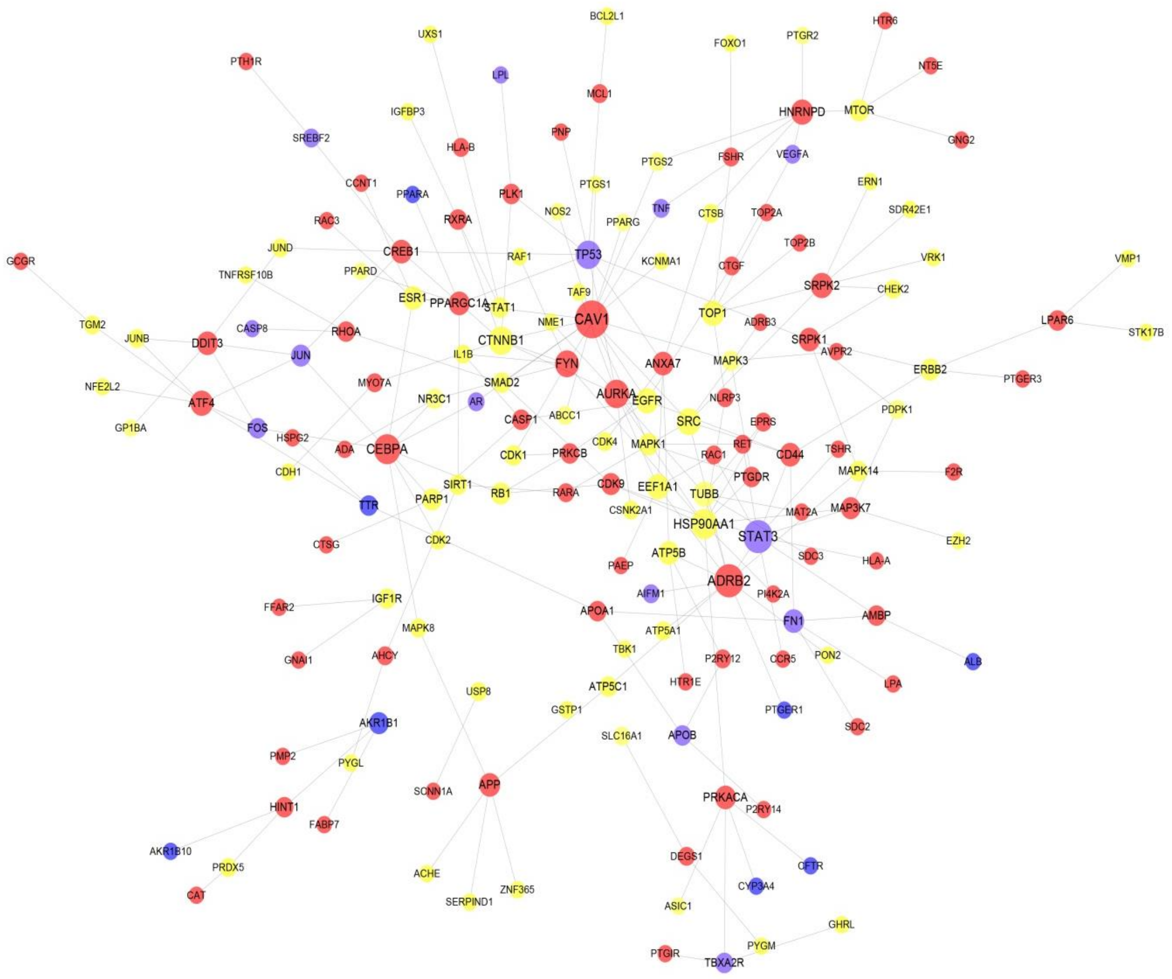

Fig S3 Protein-protein interaction analysis of formula targets and disease genes. Yellow texts represent disease genes; gray texts represent formula targets; blue texts represent the overlap between formula targets and disease genes. The network was generated in Cytoscape.

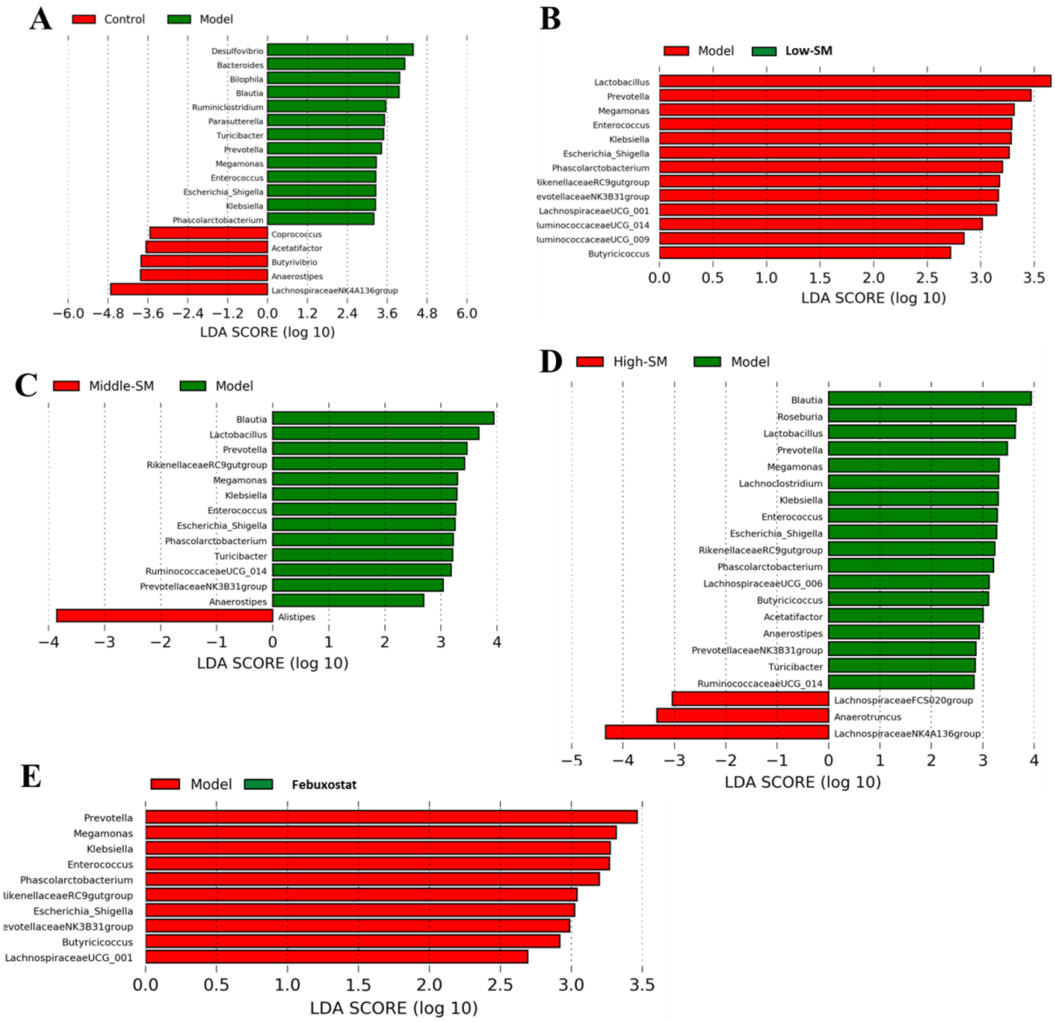

Fig S4 LEfSe identified the differential microbial genera between groups. (A) Control *vs* Model; (B) Model *vs* Low-SM; (C) Model *vs* Middle-SM; (D) Model *vs* High-SM; (E) Model *vs* Febuxostat. Significant differences are shown (LDA score >2).

p-STAT3

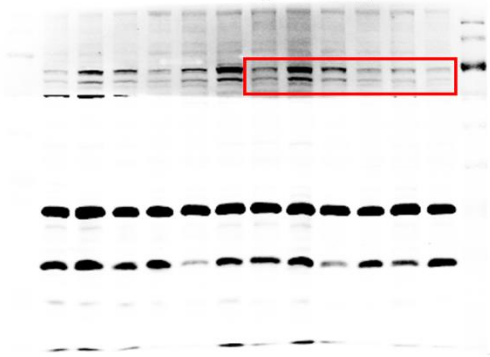

β-actin

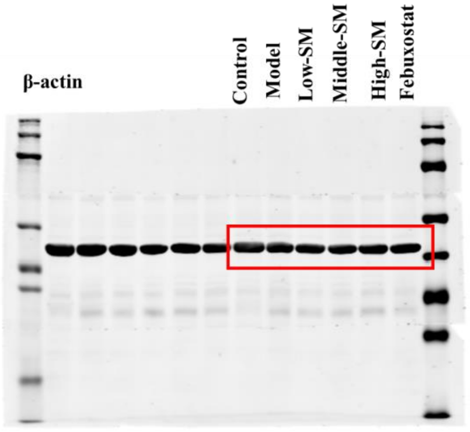

APOB

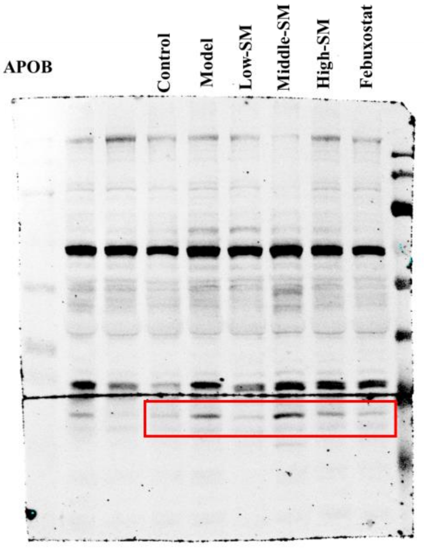

β-actin

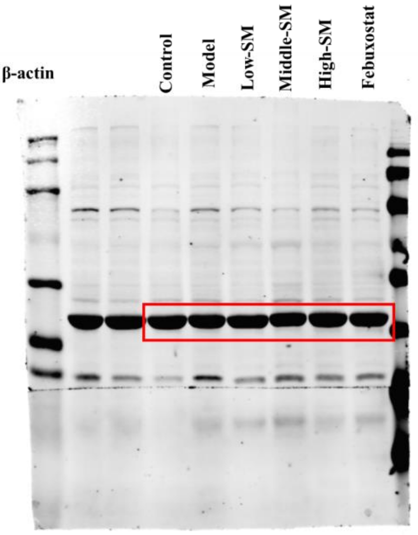

Caspase-8

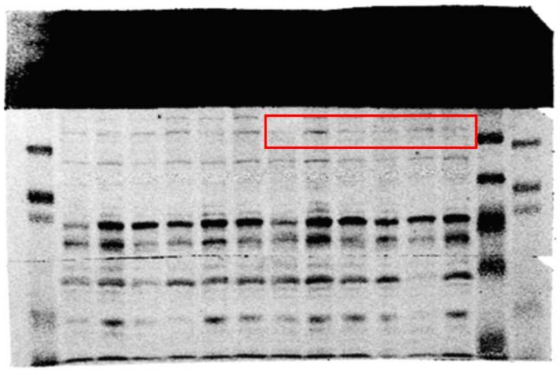

β-actin

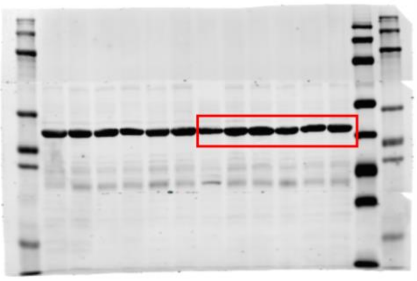

AIFM1

Control Model Low-SM Middle-SM High-SM Febuxostat

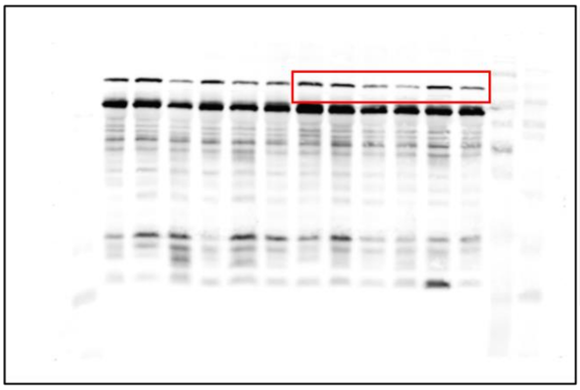

β-actin

Control Model Low-SM Middle-SM High-SM Febuxostat

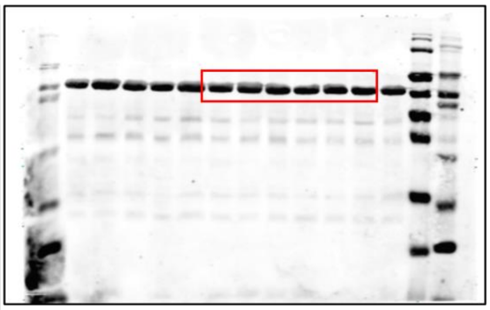

c-FOS

Control Model Low-SM Middle-SM High-SM Febuxostat

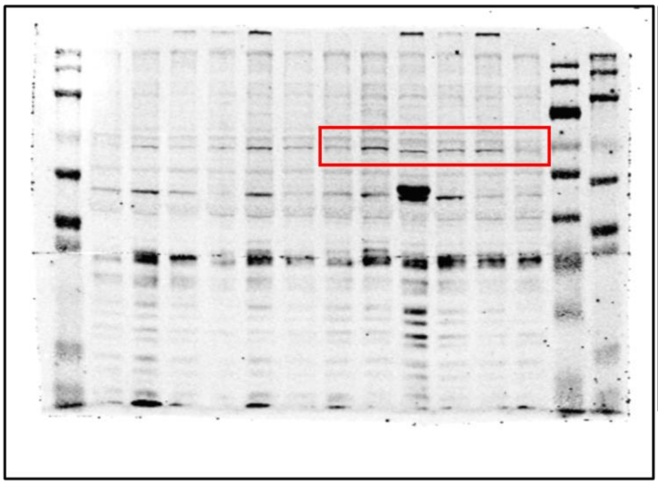

β-actin

Control Model Low-SM Middle-SM High-SM Febuxostat

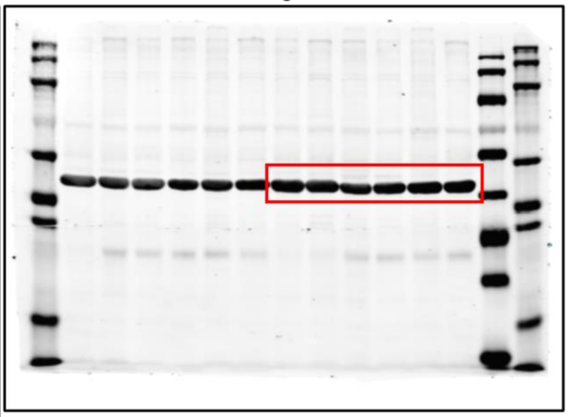

c-Jun

Control Model Low-SM Middle-SM High-SM Febuxostat

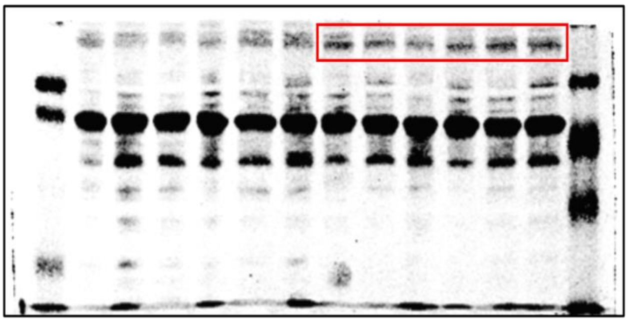

β-actin

Control Model Low-SM Middle-SM High-SM Febuxostat

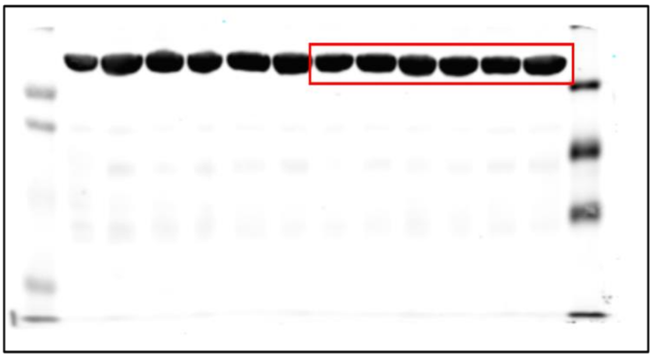

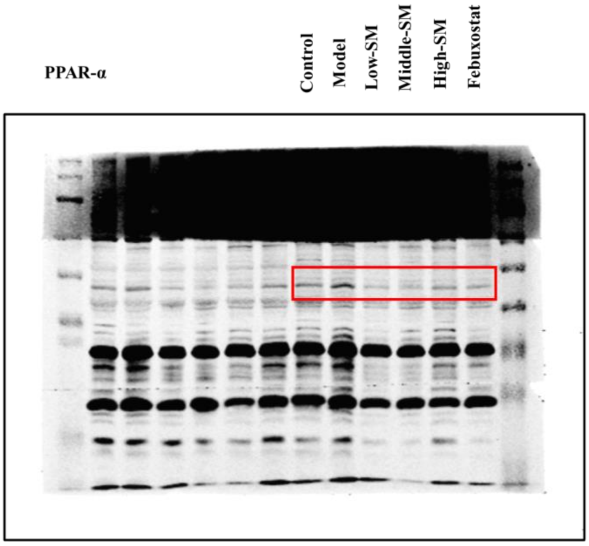
PPAR-α
Control
Model
Low-SM
Middle-SM
High-SM
Febuxostat

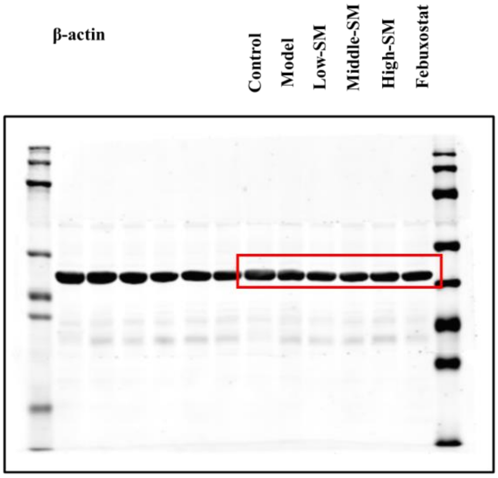
β-actin
Control
Model
Low-SM
Middle-SM
High-SM
Febuxostat

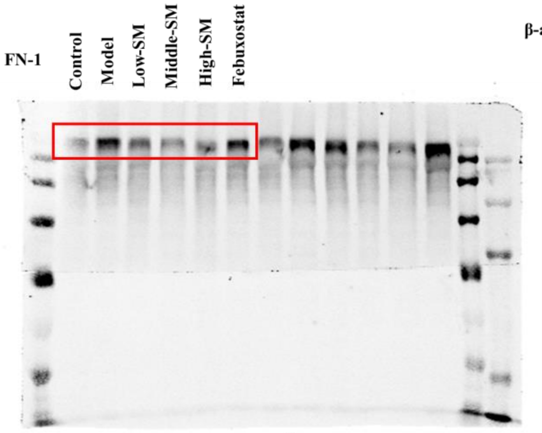
FN-1
Control
Model
Low-SM
Middle-SM
High-SM
Febuxostat

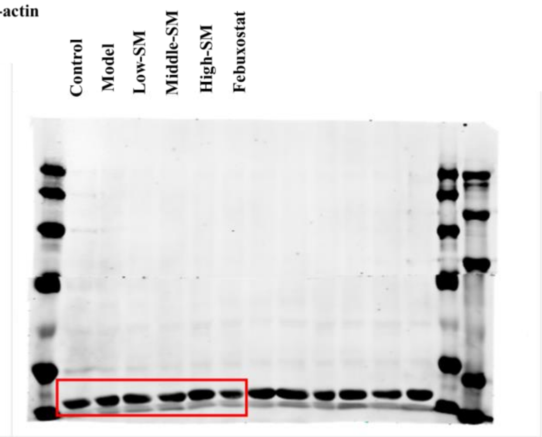
β-actin
Control
Model
Low-SM
Middle-SM
High-SM
Febuxostat

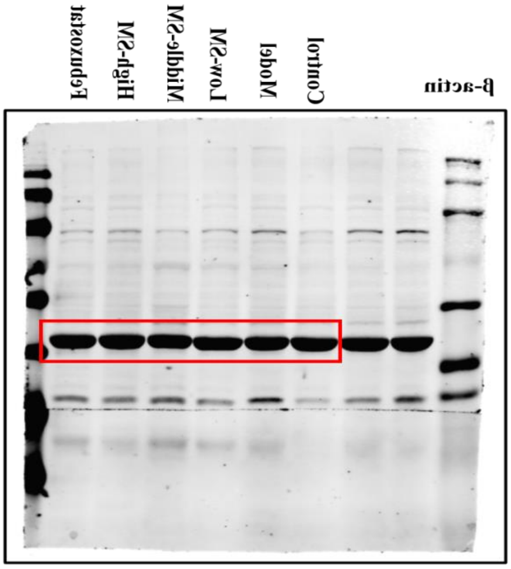
β-actin
Control
Model
Low-SM
Middle-SM
High-SM
Febuxostat

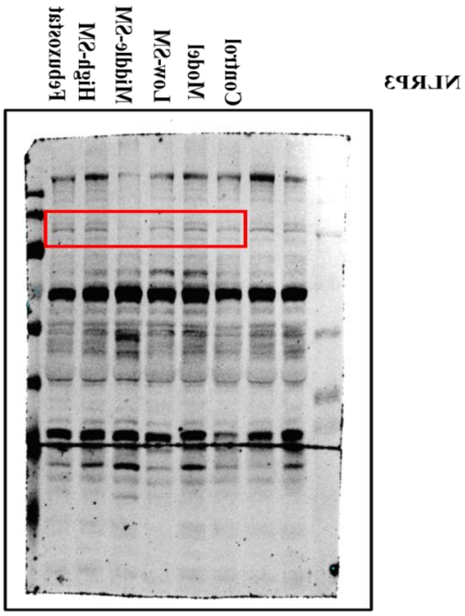
NLRP3
Control
Model
Low-SM
Middle-SM
High-SM
Febuxostat

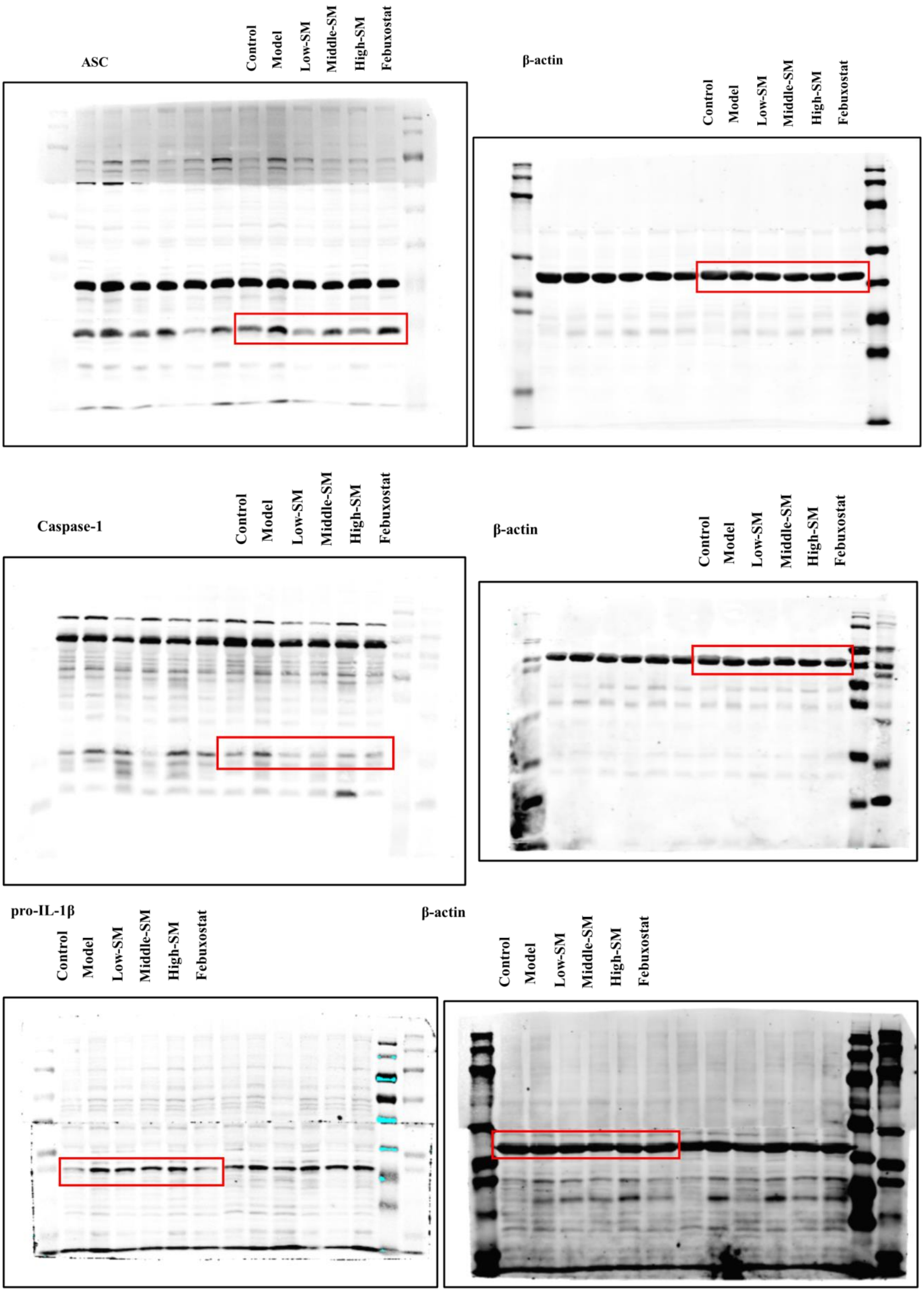

Fig S5 Uncropped images of the original western blots of p-STAT3, APOB, caspase-8, AIFM1, c-FOS, c-JUN, PPAR-α, FN-1, NLRP3, ASC, caspase-1 and pro-IL-1β.
